# ZebraZoom: an automated program for high-throughput behavioral analysis and categorization

**DOI:** 10.3389/fncir.2013.00107

**Published:** 2013-06-12

**Authors:** Olivier Mirat, Jenna R. Sternberg, Kristen E. Severi, Claire Wyart

**Affiliations:** ^1^Centre de Recherche de l'Institut du Cerveau et de la Moelle Épinière, UPMC, Inserm UMR S975, CNRS UMR 7225, Fondation ICM, Campus Hospitalier Pitié SalpétrièreParis, France; ^2^Université Paris DescartesParis, France; ^3^Université Pierre et Marie CurieParis, France

**Keywords:** machine learning, tracking, analysis of kinematics, collective behavior, support vector machine classifier, multiclass categorization, locomotion in intact behaving animals

## Abstract

The zebrafish larva stands out as an emergent model organism for translational studies involving gene or drug screening thanks to its size, genetics, and permeability. At the larval stage, locomotion occurs in short episodes punctuated by periods of rest. Although phenotyping behavior is a key component of large-scale screens, it has not yet been automated in this model system. We developed ZebraZoom, a program to automatically track larvae and identify maneuvers for many animals performing discrete movements. Our program detects each episodic movement and extracts large-scale statistics on motor patterns to produce a quantification of the locomotor repertoire. We used ZebraZoom to identify motor defects induced by a glycinergic receptor antagonist. The analysis of the blind mutant *atoh7* revealed small locomotor defects associated with the mutation. Using multiclass supervised machine learning, ZebraZoom categorized all episodes of movement for each larva into one of three possible maneuvers: slow forward swim, routine turn, and escape. ZebraZoom reached 91% accuracy for categorization of stereotypical maneuvers that four independent experimenters unanimously identified. For all maneuvers in the data set, ZebraZoom agreed with four experimenters in 73.2–82.5% of cases. We modeled the series of maneuvers performed by larvae as Markov chains and observed that larvae often repeated the same maneuvers within a group. When analyzing subsequent maneuvers performed by different larvae, we found that larva–larva interactions occurred as series of escapes. Overall, ZebraZoom reached the level of precision found in manual analysis but accomplished tasks in a high-throughput format necessary for large screens.

A central question in systems neuroscience is how neural circuit assembly and function relate to animal behavior. Genetic screens in invertebrate models, such as *Drosophila melanogaster* and *Caenorhabditis elegans* have begun to unravel the genetic basis of circuit function and behavior (Chalfie et al., 1985; Moore et al., 1998; Scholz et al., 2000). Automated methods have recently been developed in these species to track the position of individuals alone or in a group (Branson et al., 2009; Swierczek et al., 2011) and to categorize behavior (Dankert et al., 2009; Kabra et al., 2013). The zebrafish has emerged as an important vertebrate model organism for developmental biology, neurobiology, and human disease models, and is now used as a genetic model organism for the study of the mechanisms modulating complex behaviors in vertebrates such as depression and anxiety (Blaser et al., 2010; Lee et al., 2010; Cachat et al., 2011; Vermoesen et al., 2011; Zakhary et al., 2011; Ziv et al., 2013), sleep (Zhdanova et al., 2001; Appelbaum et al., 2009), or addiction (Petzold et al., 2009; Khor et al., 2011). The permeability, small size, genetic tractability, transparency, and low cost of zebrafish make them highly suitable for large-scale genetic and chemical screens (Driever et al., 1996; Granato et al., 1996; Haffter and Nusslein-Volhard, 1996).

Although simple for a vertebrate, the locomotor patterns of the zebrafish larva bring technical challenges to automated analysis. Larvae spontaneously swim in discrete bouts in a manner often described as “beat and glide,” which can be classified as individual maneuvers, including slow forward swim, routine turn, or escape. These short movements are characterized by a large range of tail-beat frequencies (15–100 Hz), which require high-speed imaging to capture accurately and can be separated by long resting periods of up to a few seconds. Manual tracking via frame-by-frame analysis has formed the basis of contemporary knowledge and has enabled initial characterization of the larval zebrafish locomotor repertoire (Budick and O'Malley, 2000; Borla et al., 2002; McElligott and O'Malley, 2005). However, manual techniques are both laborious and limited in scope for high-throughput screens (Driever et al., 1996; Granato et al., 1996; Haffter and Nusslein-Volhard, 1996). The currently available automated tools have limitations in either refinement or time-scale. Recent chemical or genetic screens have relied on commercial software that estimates an index of mobility of the larvae, usually measured as the distance traveled during a recording session or the amount of time spent moving (Rihel et al., 2010; Elbaz et al., 2012; Rihel and Schier, 2012). These approaches for high-throughput screens provide information about average velocity and distance traveled by tracking the animals' center-of-mass over minutes to hours. Previous studies have either focused on analyzing movement duration and speed at low frequency over long periods of time or on fine analysis of kinematics at high frequency but for very short acquisition (typically 1000 ms, Burgess and Granato, 2007; Liu et al., 2012). Accurate categorization of maneuvers for each individual in a group requires novel methods to record behavior with high temporal resolution and over long durations, automatically tracking and categorizing thousands of maneuvers.

Here we developed a new program, ZebraZoom, to track the full body position over a multiple-minute timescale of 56 larvae simultaneously recorded at high frequency and to finely characterize each maneuver. To identify core and tail positions for large datasets, videos were obtained on multiple larvae simultaneously over long periods of time and at high resolution using a high-speed camera run in a streaming-to-disk interface (Methods). Typically 500–1000 movements from seven larvae were recorded per dish in four minutes and eight dishes were monitored in parallel. To simplify tracking, we placed larvae in conditions that reduced overlapping in the z-plane during swimming (Methods; overlaps occurred on average once every 145 s per larva). We developed an offline 2D tracking method for identifying and separating each animal even when in close contact (Methods, Figure 1). For each larva several features were identified, a core position that included the head and swim bladder (Figure 1A) and ten points along the tail (Methods and Figure 1B; Video S1). As movements occurred as discrete episodes, ZebraZoom detected movements based on the tail-bending angle over time (Methods and Figures 1C–D). To validate the accuracy of movement detection, one trained experimenter manually identified all movements occurring in a subset of videos. In three videos representing a total of 189 events, movements occurred with a false negative rate of 2.7% and a false positive rate of 3.7%.

**Figure 1:**
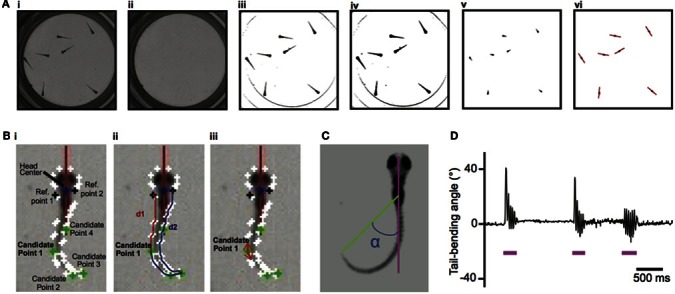
**Image processing for tracking of larvae's core positions and larvae's tail and detection of movements based on the tail-bending angle. (A)** Tracking of the larvae's core positions. **(Ai)** Initial image. **(Aii)** Background image. **(Aiii)** Image with background subtracted. **(Aiv)** Binary image. **(Av)** Eroded image. **(Avi)** For each larva, identification of the core (blue dot) and heading direction (red axis). **(B)**. Identifying the tip of the tail. **(Bi)** The head center is located at the boundary of the head and trunk. Candidate Point 1–4 along the tail are the four points of the contour with the smallest *x*-value, smallest *y*-value, largest *x*-value, and largest *y*-value caudal to reference points 1 and 2. **(Bii)** The two distances d1 and d2 shown for candidate point 1. **(Biii)** The two vectors used to identify the tail tip defined with the minimal scalar product for candidate point 1. **(C)** Definition of the tail-bending angle (α) separating the body axis (pink) and the line connecting the core and the tip of the tail (green). **(D)** Example of the tail-bending angle over time with detection of movements indicated by the pink line.

To quantify movements in a consistent manner, we used the location of the head, the position of the tail, the heading direction and the tail-bending angle to estimate global parameters of locomotion (Figure 2A, Methods). We observed that movements for 5–7 dpf wild-type (WT) larvae occurred every 2.22 s on average per larva (at 0.4495 ± 0.0117 Hz). For all movements identified, larvae performed on average 3.19 ± 0.01 oscillations per movement, had a 24.29 ± 0.03 Hz tail-beat frequency (TBF), lasting 189.5 ± 0.0004 ms with a 51.14 ± 0.18° heading direction range, 2.49 ± 0.008 mm traveled distance and 13.35 ± 0.04 mm/s speed per maneuver. We illustrated the use of ZebraZoom for quantifying the effects of a known glycinergic receptor antagonist, and for analyzing a blind genetic mutant. Glycine is responsible for reciprocal inhibition in the spinal cord that permits left-right alternation to sustain oscillations (Dale, 1985; Grillner et al., 1995; Granato et al., 1996; Drapeau et al., 2002; Li et al., 2004). In zebrafish, mutants for glycinergic receptors or transporters have been associated with defects in motor pattern generation (Granato et al., 1996; Odenthal et al., 1996; Hirata et al., 2005; Masino and Fetcho, 2005). We measured the effect of bath application of 75 μM strychnine on spontaneous locomotor activity in larvae and compared to control siblings that were not exposed to the drug (Figure 2B, Methods). For control larvae, we did not observe a significant change in the occurrence of movements over time (0.35 ± 0.05 movements per larva/s before and 0.27 ± 0.03 movements per larva/s after), or on any of the global parameters (Figure 2B; all *p* > 0.15). However the locomotor behavior of larvae treated with strychnine was significantly impacted (Figure 2B). Overall, movements occurred less frequently (0.30 ± 0.04 Hz before and 0.12 ± 0.02 Hz after, *p* < 0.0002). Although the average TBF during a movement did not change (*p* > 0.81), the number of oscillations decreased (3.52 ± 0.17 before and 2.89 ± 0.16 after; *p* < 0.0078), an effect that was associated with a decrease in movement duration (*p* < 0.0001), distance traveled (*p* < 10^−5^), and average speed (*p* < 10^−5^). Strychnine application also resulted in a decrease in the range of heading direction (*p* < 10^−5^). *atoh7* mutant larva lack retinal ganglion cells, rendering them blind (*atoh7*^−/−^, Kay et al., 2001). Considering the importance of vision for zebrafish larvae, analyzing their locomotor output could reveal corresponding behavioral differences. Overall *atoh7*^−/−^ mutants generated episodic movements less frequently than control siblings (0.33 ± 0.02 Hz vs. 0.51 ± 0.02 Hz, 112 larvae for each condition). Quantitative analysis of global parameters of the blind mutants showed no difference in the average TBF or the average speed per larva (Figure 2C; *p* > 0.85 and *p* > 0.83, respectively) but there were small but significant decreases in the number of oscillations, duration, heading direction range, and distance traveled (Figure 2C; all *p* < 10^−3^). These defects were observed systematically in four clutches. *atoh7*^−/−^ mutants thus display small but substantial differences in basic motor behavior when compared to control siblings.

**Figure 2:**
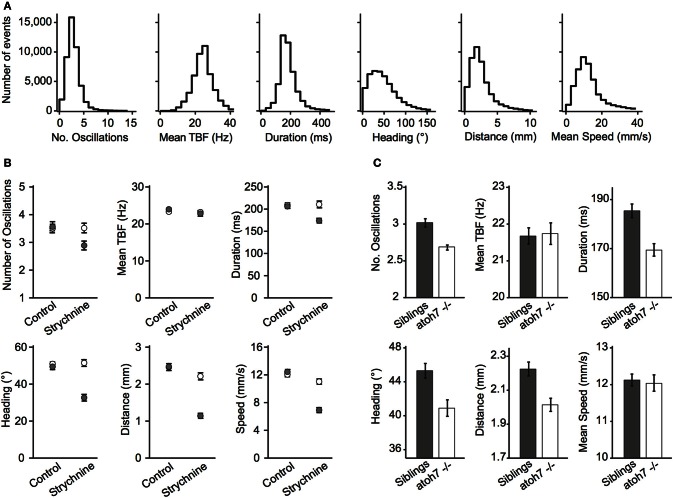
**Global parameters describing locomotion in wild-type, mutant, or drug-treated larvae. (A)** Distribution of global parameters of movements for 5–7 dpf WT larvae. From left to right: Number of oscillations per movement, TBF in Hz across all movements, duration of each movement in ms, heading direction range in degrees, distance traveled per movement in mm, speed in mm/s during each movement (eight videos, 420 larvae, six clutches, 5–7 dpf, 44,688 movements). All values were calculated per movement. **(B)** Effect of the glycinergic receptor antagonist strychnine on the global parameters of movements. White circles are before application, gray are after application (two videos, 42 larvae for each condition, two clutches, 6–7 dpf, 10,459 movements). **(C)** Effect of the *atoh7* mutation on the global parameters characterizing movements (four videos, 112 mutants *atoh7*^−/−^, and 112 control siblings, four clutches, 6 dpf). For **(B,C)**: error bars are standard errors of the mean and statistics were calculated per larva.

Zebrafish larvae display a variety of locomotor maneuvers that are often grouped into discrete categories. In these experimental conditions, three types of movement occur in groups of larvae at early stages: slow forward swims (S), routine turns (T, also referred to as slow turns), and escapes (E, including C-turns or burst swims). Figure 3 shows examples of these movements reported by ZebraZoom. For each maneuver, we superimposed a succession of images (Figures 3Ai–Ci), the tail-bending angle over time (Figures 3Aii–Cii) and the curvature along the rostro-caudal axis and as a function of time (Figures 3Aiii–Ciii). The three types of maneuvers included a series of slow left-right alternation; high values of curvature were confined to the caudal tail (Figures 3Aiii–Ciii). While high values of curvature of the tail were confined to the caudal end for slow forward swims (Figure 3Aiii), high values of curvature were distributed from head to tail for routine turns and escapes (Figures 3Biii,Ciii). Stereotypical routine turns and escapes differed by the frequency of left-right alternation in the tail bend (Figures 3Biii,Ciii). As larvae did not always exhibit a canonical slow forward swim, routine turn or escape, some movements were ambiguous. To estimate the percentage of these movements, four experimenters subjectively classified 390 movements distributed over eight videos. Overall about 82% of all movements were classified uniformly by at least three out of four experimenters (Methods) indicating that 18% of movements were difficult to categorize.

**Figure 3:**
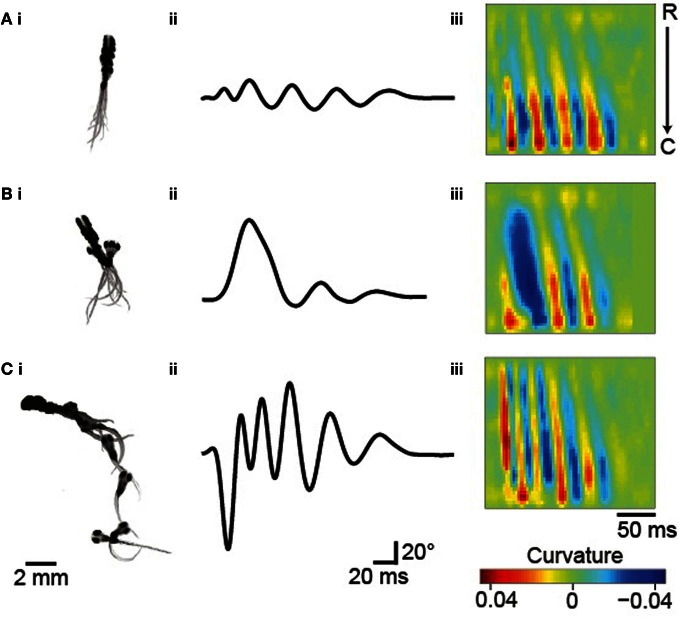
**Typical maneuvers occurring in groups of 5–6 dpf larvae. (A)** Slow forward swim (S). **(B)** Routine turn (T). **(C)** Escape response (E). **(Ai–Ci)** Superimposed images taken every 17 ms. **(Aii–Cii)** The tail-bending angle over time for each maneuver. **(Aiii–Ciii)** Plots of the curvature of the tail as a function of time and position along the rostro-caudal body axis.

Using knowledge of stereotypical locomotor events, we designed a multiclass categorization approach with supervised machine learning to automatically sort each movement into one of the three categories. To implement the multiclass categorization, we used two successive support vector machine (SVM) classifiers: the first classifier sorted S vs. all other maneuvers, and when necessary the second classifier sorted T vs. E. Locomotor events were segregated subjectively in the training set (*n* = 201). This machine learning approach relied on associating dynamic parameters extracted from the tail-bending angle over time with each maneuver type identified in the training set (Figure 4A and Methods). To reduce the dimensionality of the data, we performed Principal Component Analysis (PCA). Based on the selection of a trained experimenter on the learning set, we validated the multiclass categorization to sort maneuvers by comparison with the subjective classification performed by a trained experimenter for a recognition set (*n* = 189; Figures 4B,C). We observed that ZebraZoom agreed with the trained experimenter 82.5% of the time for the recognition dataset (85% for S, 82% for T, and 79% for E; Figures 4B,C; Table 1 and Methods). When compared to four independent experimenters, ZebraZoom reached 91% accuracy for categorization of stereotypical maneuvers that all experimenters had unanimously identified and 76.4% on average for all maneuvers (73.2–82.5%, Table 1). Once validated, we applied the ZebraZoom categorization algorithm on a large dataset of 44,688 movements of WT larvae (Figure 4D). We identified 14.911 S (33.36%), 21,432 T (49.96%), and 8,345 E (18.67%). The distribution of global parameters for the three classes of maneuvers were similar in terms of number of oscillations and duration, but they differed in terms of mean TBF, heading direction range, distance traveled and speed (Figure 4D).

**Figure 4:**
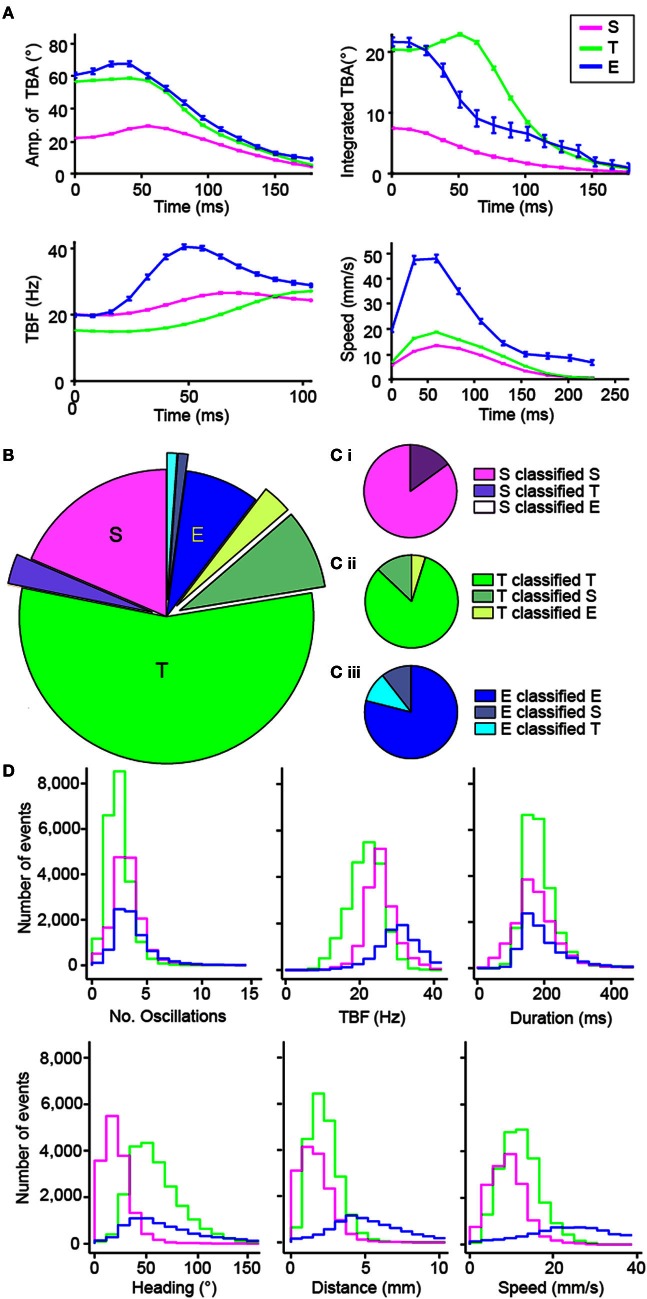
**Validation of the automated categorization of maneuvers: slow forward swim (S), routine turn (T) and escape (E). (A)** Dynamic parameters used for categorizing the different maneuvers: amplitude of tail-bending angle (TBA) in degrees, integrated TBA in degrees, TBF in Hz, and speed in mm/s. The mean of each parameter for each time bin is shown and error bars are standard error of the mean: S in pink, T in green and E in blue. Time 0 is taken at the peak of the first bend of the movement. **(B,C)** Comparison of the results of the automatic categorization from ZebraZoom with the subjective categorization by a trained experimenter on 189 movements from one video. The comparison of the categorization is shown overall **(B)** and for each maneuver (**C**: **Ci** for S, **Cii** for T, **Ciii** for E). The proportion of movements categorized the same way by both methods is shown in addition to the proportion of movements miscategorized and how they were categorized. **(D)** Distribution of global parameters for each maneuver S, T, and E of WT larvae (same color code as in **A**; 44,688 movements total from eight videos, six clutches).

**Table 1:** **Estimation of ZebraZoom categorizing accuracy based on the different reference experimenters**.

	**All movements (%)**	***S* (%)**	***T* (%)**	***E* (%)**
Experimenter #1	82.5	85	82	79
Experimenter #2	73.2	85	60	53
Experimenter #3	74.3	73	80	58
Experimenter #4	75.4	79	77	47

The investigation of interactions between individuals leading to coordinated motion in animal groups has been a long-standing challenge that is central to elucidating the mechanisms and evolution of collective behavior. Most studies have focused on the analysis of speed or directionality to reflect the interaction between animals (Katz et al., 2011; Gautrais et al., 2012). We availed ourselves of ZebraZoom's features to accurately identify each larva and categorize their maneuvers to study how larvae interacted. In comparison to juvenile and adult zebrafish that swim continuously, larval zebrafish swim episodically with maneuvers that occur in a beat-and-glide manner. Each movement can be regarded as a discrete event, therefore we were motivated to explore how local perturbations of a single individual could impact the group. The program switched identity of larvae once every 109 s (once every 49 movements on average), allowing us to track single larvae. We modeled sequences of maneuvers performed by larvae within a group as Markov chains. Utilizing the classifier, we described larva–larva interactions in a group and intrinsic properties of individuals. We calculated a transition index (*I*) for each sequence of two maneuvers as the transition probability between first and second maneuvers divided by the probability of random occurrence of the second maneuver (Figure 5; Table 2 and Methods). When the two successive maneuvers were the same, a higher transition index indicated the probability of repetition of this maneuver was greater than chance. The transition index was equal to one when the order of sequential maneuvers was random. Overall *I* was greater than one for repetition of the same maneuvers (Table 2). We sorted the data into interactions between different animals and the repetition within the same animal. We analyzed how the transition index for a given succession of maneuvers depended on the distance between the two larvae's core positions at the onset of the movement and the time between the onset of each movement (Figure 5 and Methods). Individual larvae often performed the same type of maneuver sequentially (maximal values *I*_S max (same)_ = 1.43, *I*_T max (same)_ = 1.37, *I*_E (same)_ = 2.38, all *p* < 0.002; Figure 5A, Table 2, and Methods). Although slow forward swims or routine turns were not frequently repeated between larvae (*I* close to 1: maximal values *I*_S (diff)_ = 1.09 and *I*_T (diff)_ = 1.01, *p* > 0.05; Figures 5Bi,ii, Table 2, and Methods), we found that recurrent escapes were very frequent between different larvae (maximal value *I*_E (diff)_ = 3.6, *p* < 0.002; Figure 5Biii, Table 2, and Methods). Five to seven dpf larvae do not show evidence for social interactions (Buske and Gerlai, 2011). By taking advantage of the algorithm for identifying single larva and categorizing simple maneuvers, we reveal that larva–larva interactions primarily occurred for escape responses. These series of escapes occurred after direct collisions (in one third of the cases) or via long distance interaction (two third of cases). Blind *atoh7*^−/−^ larvae showed a similar profile of interactions for escapes (data *not shown*); these interactions were most likely mechanically triggered.

**Figure 5:**
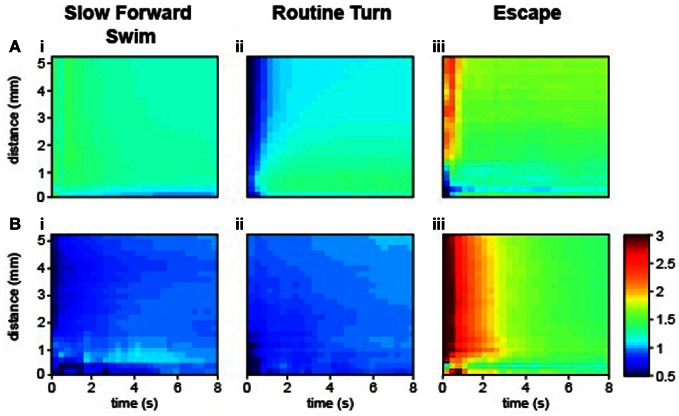
**Larva–larva interactions occurred most frequently as sequences of escapes.** Transition index for the same larvae **(A)** and for different larvae **(B)**. Time is plotted in seconds from the time of initiation of the first movement. Distance is plotted from the core position of the larva at the beginning of a movement. **(Ai,Bi)** The sequence S–S. **(Aii,Bii)** The sequence T–T. **(Aiii,Biii)** The sequence E–E (36,068 total movements from five videos, 280 WT larvae, four clutches, 5–6 dpf).

**Table 2:** **Transition index for sequence of two maneuvers estimated as the probability of transition from maneuver 1 to maneuver 2 divided by the occurrence of the maneuver 2**.

	***S***	***T***	***E***
**INTERACTIONS BETWEEN DIFFERENT ANIMALS**			
S	1.0498	1.0152	0.8481
T	1.0087	1.0413	0.8606
E	0.8549	0.8493	1.7528
**REPETITIONS WITHIN THE SAME ANIMAL**			
S	1.2162	0.8947	0.8413
T	0.9091	1.1185	0.8499
E	0.8263	0.8887	1.6995

Large-scale chemical and genetic screens would benefit from a quantitative approach to analyze fine locomotor patterns over long periods of time. Compared to other genetic models, zebrafish locomotion is difficult to analyze because larvae initiate maneuvers intermittently and during these short events, the larvae swim at a high speed with TBFs ranging from 15–100 Hz. The quantitative analysis of motor behavior for large-scale screens requires solving the problem of recording multiple animals simultaneously at high frequency (above 200 Hz) and for long periods of time (minutes). Here we implemented a reliable method for quantifying global parameters of movements based on stream-to-disk recordings acquired at high frequency and over long periods of time, limited only by data storage. Next we developed a robust method for tracking the full body position of zebrafish larvae swimming in groups. We first manually validated that the tracking accurately detected discrete movements, and then used the global parameters obtained to characterize the locomotion of WT larvae. Quantification of the global parameters describing larval movements corroborates previous observations based on fewer samples (Budick and O'Malley, 2000; Danos and Lauder, 2007; Liu et al., 2012). Similar estimates of the duration of movements, distance traveled and speed were obtained from the recent application of C-trax (designed originally for *Drosophila*) to zebrafish larvae [Lambert et al. (2012) based on Branson et al. (2009)]. In these conditions, recordings at low frequency over long periods of time, typically 60 Hz for minutes or hours, revealed the global level of activity over time but no information on fine kinematics during individual maneuvers (Elbaz et al., 2012). When recordings were performed at high frequency to capture the dynamics of motion, they usually lasted 1000 ms (Burgess and Granato, 2007).

We illustrated the benefit of ZebraZoom to quantify global parameters of movements by analyzing the effect of a drug to block glycinergic neurotransmission, which has been known to be involved in motor pattern generation and alternation between the left and right side of the spinal cord across vertebrate species (Grillner, 2003; Korn and Faber, 2005; Nishimaru and Kakizaki, 2009). Most studies relied on ventral nerve root recordings where muscles were dissected out or paralyzed in order to record the activity of motor neurons at the level of a few segments at most. Our automated quantification of locomotor events enabled identification of effects induced by bath application of the glycinergic antagonist strychnine on locomotion in intact animals. As predicted, bath application of strychnine dramatically reduced the occurrence of movements and the number of oscillations per movement, that was correlated with a reduction of the duration of movement and of the distance traveled. While mean TBF was not affected, we observed a reduction in the heading direction range and in speed. Our approach pinpointed effects of glycinergic blockade, including a reduction in the number of oscillations per movement, a kinematic feature not estimated in commercially available software. The analysis of the mutant *atoh7* revealed that although TBF and speed were not affected in the blind mutant, there was a small but significant decrease in the number of oscillations, heading direction range, distance, and duration of each bout compared to their control siblings. These effects were systematically observed on four clutches suggesting that visual feedback may impact some global parameters of locomotion. However since the pattern of expression of *atoh7* has not yet been fully characterized, it cannot be excluded that the gene may be expressed in cells other than retinal ganglion cells.

The originality of ZebraZoom lies in categorizing all maneuvers performed by individual larvae in a group. The subjective analysis of maneuvers based on four independent experimenters revealed that locomotor maneuvers were not obvious to categorize. Based on subjective estimates, 18% of all movements corresponded to ambiguous maneuvers. By using a machine-learning paradigm, we trained ZebraZoom to categorize all maneuvers over tens of thousands of movements with 82.5% accuracy, a similar value to the 72% agreement rate of all four experimenters measured over a few hundreds of movements. The approach we developed here could be expanded to include directionality of the turns, sequences of maneuvers such as those occurring during prey tracking, and subcategories of escapes.

This study constitutes an important first step for accurate tracking of multiple larvae in groups over long periods of time and for categorizing maneuvers. Some improvements could be implemented in the future. While our tracking method currently relies on a simple “blob” approach solely based on raw image analysis, a model-based approach may be more reliable in particular when animals are in close contact (Fontaine et al., 2008). We show here that ZebraZoom can achieve an accurate categorization of maneuvers, comparable to experimenters' estimates, based solely on the dynamics of movement of head and tail. An interesting avenue of exploration to address this could be investigation of novel dynamic parameters for the learning and recognition process of the classifier to yield subtler methods for detection of defects. Quantification of motor patterns in *C. elegans* is based on a description of all possible positions of the animal over time (Stephens et al., 2008). In order to fully understand larval zebrafish behavior we need to identify a minimal set of parameters sufficient to describe all motor patterns. All together this work brings new insight to the complexity of behavior determination in zebrafish larvae and could be applied to investigation of the mechanisms of addiction, arousal, feeding, social interaction and aggression in larvae and juveniles (Gahtan et al., 2005; Bianco et al., 2011; Buske and Gerlai, 2011; Miller and Gerlai, 2012; Ziv et al., 2013). The observation of complex interactions in juveniles raises the hope that it will soon be possible to investigate the neuronal circuits and molecular pathways underlying social interactions. The fact that we can track individual larva and analyze their interactions is a major advance over existing methods. Our approach that systematically quantifies and categorizes thousands of motor patterns was designed to bring efficiency and reliability to drug screening and forward genetic screens. ZebraZoom can detect, quantify, and categorize movements to provide a quantitative description of global parameters as well as a qualitative description of all maneuvers performed by individual larvae.

## Methods

### Zebrafish husbandry

All experiments were performed on *Danio rerio* larvae between 5 and 7 dpf. AB and TL strains of WT larvae were obtained from our laboratory stock of adults. Embryos and larvae were raised in an incubator at 28.5°C under a 14/10 light/dark cycle (lights on, 8:00 A.M.; lights off, 10:00 P.M.) until the start of behavioral recordings. The mutant line for *atoh7* (Kay et al., 2001) was given by Dr. Herwig Baier, MPI Munich. Double recessive *atoh7*^−/−^ mutants were identified at 5 dpf by their dark pigmentation. All procedures were approved by the Institutional Ethics Committee at the Research Center of the Institut du Cerveau et de la Moelle épinière (CRICM).

### Behavioral recordings

Motor behavior of 56 larvae split into eight dishes (seven larvae per dish, Figure 1Ai) on a homogeneous illumination plate (light intensity 0.78 mW/cm^2^, Phlox, ref. LEDW-BL-200/200-LLUB-Q-1R24) in egg water (http://zfin.org/zf_info/zfbook/chapt1/1.3.html, methylene blue added at 0.5 ppm). Following acclimation, larvae were recorded for 4 min at 337 Hz with a high-speed camera (VC-2MC-M340E0-C, CMOS chip 2048 × 1088 pixels, Vieworks, South Korea) placed above the setup and coupled to a camera objective (AF Nikkor 50 mm f/1.8D, Nikon, Japan). Pixel size was 66 μm. We developed a direct-to-disk high-speed imaging system designed for long acquisitions of raw images in collaboration with R&D Vision, France. Behavioral recordings were performed between 2:00 and 5:00 P.M. Larvae were acclimated for 60 minutes on the light source at room temperature (21–22°C) and kept at room temperature during all recordings. Larvae were kept in dishes with an inner diameter of 2.2 cm and an outer diameter of 3.5 cm (Figure 1A). Water was kept at a low level (2 mm) in order to reduce the occurrence of crossings between larvae. Typically 500–1000 movements were recorded in each 4-min session for each well.

### Zebrazoom tracking algorithm

The first step is to track the core and then the tail for all larvae over time. Written in C++ using the *openCV* library, the program identified the center position and heading direction of each larva (Figures 1Ai–vi). The algorithm used a Hough transform to identify the eight wells. For each well the background was estimated as the maximum pixel value over all frames of the video recording (Figure 1Aii) and then subtracted for all frames for that well (Figure 1Aiii). The resulting image was converted to binary (Figure 1Aiv). An erosion filter was applied twice in a row with a 3 by 3 structuring element (Figure 1Av). The “core” of the larva referred to the resulting connected components that had an appropriate area (between 0.0871 and 0.8712 mm^2^). The core of the larva included the head and the trunk with swim bladder (Figure 1Avi). The algorithm identified the head center position as the center of mass of the putative cores for each larva in a frame. To follow each larva across subsequent frames, ZebraZoom used the information from the previous two frames (core position and speed) to predict the position of the larva and located the closest core out of all the possible cores. The heading direction for each larva was calculated simultaneously using the moments of the eroded body (up to the second order, see red lines in Figure 1Avi).

For each larva with an identified core, we determined the “full body” referring to the connected component of the binary image in Figure 1Aiv. In order to track the tail, the full body was rotated so that the head axis was parallel to the y-axis, always in the same orientation. To identify the contour of the tail in the coordinate system defined by the head axis, a series of points was extracted from the full body by using the algorithm of Suzuki and Abe (1985), (white dots in Figures 1Bi–iii). Reference point A1 was the closest point on the contour line from the head center and reference point A2 was the point symmetrical along the head axis to reference point A1 on the contour. In order to identify the tip of the tail, four candidate points on the contour were selected with minimal and maximal *x*- and *y*-values (Figures 1Bi–iii). For the maximal *y*-value the point also had to be above a given distance away from the two reference points [below a 20% threshold for the ratio |(*d*_1_ − *d*_2_)|/(*d*_1_ + *d*_2_), Figure 1Bii]. Distances d1 and d2 were calculated from each candidate point to the reference points A1 and A2 along the contour (Figure 1Bii). Candidate points with a ratio |(*d*_1_ − *d*_2_)|/(*d*_1_ + *d*_2_) over 0.25 were excluded. The tip of the tail was then identified as the point associated with the smallest scalar product of the tangential vectors pointing in opposite directions (Figure 1Biii). The midline of the larva was defined as the line equidistant to the contour line on the left and right side.

### Errors in core and tail tracking

If an error occurred in the core tracking, the larva was missing for that frame and there was no tracking of its tail. If the core of a larva was identified, the algorithm proceeded to the tail tracking. To confirm that the tail tracking was correct, the algorithm checked that the tail length was greater than 1.32 and less than 3.96 mm. If this criterion was invalid, the tail position was set to the previous frame. This happened in 13.46% of frames on average but was compensated by a smoothing spline on the center positions between the left and right contour points of the tail and a median filter applied on the tail-bending angle over time. The tail-bending angle was defined as the angle between the axis formed by the tip of the tail and the center of the head with respect to the larva heading direction (Figures 1C,D).

### Separating larvae during contacts

Tracking was optimized to separate larvae in close vicinity to one another or in direct contact. For core tracking, if the trajectories of the two cores merged at a given time point, then the algorithm considered that a collision occurred between the two larvae. When the predicted positions of two larvae based on core position and speed in the two previous frames were closest to the same core, the algorithm considered that a collision between the two larvae occurred at that frame. When a collision was detected, the algorithm applied erosion filters in the region of interest defined by the core until more than one isolated core emerged. In rare cases, the multiple cores were not resolved and the larva could not be tracked for that frame. For the tail tracking, if the area of the larva's full body was greater than 1.9 mm^2^, the algorithm considered that two larvae were in direct contact. The distance separating the larvae's cores determined which of two algorithms was used to isolate the tails: if the distance was less than 1.32 mm, a line separation algorithm was applied. A line was created to separate the two larvae by optimizing the area of the resulting tails, calculated by maximizing the sum of the two largest areas containing a head center position. If the distance was greater than 1.32 mm, a pixel intensity separation algorithm was applied instead. The threshold used to convert the image into binary was adjusted until two separate full bodies, each a connected component, emerged and contained the head center position. Larvae crossings occurred once every 145 s on average per larva (0.0069 ± 0.0019 events per second) and the switching of identification between two larvae after a collision was estimated manually to occur every 109 s on average (0.0092 ± 0.0036 events per second per larva based on 720 s of recordings from four videos, and 28 larvae).

### Detection of movements

Algorithms for the detection of movements and the behavior analysis were written in MATLAB (The Mathworks, Inc., USA). The detection of movement was based solely on thresholding the tail-bending angle measured over time (Figures 1C,D). ZebraZoom detected the start of a movement when the value for the tail-bending angle at a given frame varied over 1.15° from the mean value of the tail-bending angle for the ten surrounding frames, or 29.7 ms. To avoid separating single maneuvers into multiple events, movements that occur within 14.8 ms of each other were merged. To avoid false positives we considered only movements in which the larva core had moved more than 0.099 mm and where the range of tail-bending angle values was above 2.86 degrees. Additionally, only events during which the eroded binary image of the larva had moved more than a set number of pixels between subsequent images were considered based on the parameters used for the erosion. Rarely we have observed two distinct movements occurring without a pause, such as a slow forward swim followed by an escape due to a collision. In these few cases when two movements occurred without a noticeable stabilization in the tail-bending angle over time, the movements were merged into one movement in our analysis.

Our tracking method was robust in these experimental conditions. We cannot probe the impact of a reduction of contrast or spatial resolution. All numerical thresholds used above for tracking were fixed empirically, but they could easily be modified for other users to adapt to other recording conditions.

### Calculation of the curvature

After alignment of the body axis with the y-axis in a consistent orientation, the tail was represented parametrically in Cartesian coordinates as [*x*(*t*), *y*(*t*)]. The midline of the tail was fitted to the *x*(*t*), *y*(*t*) function with a spline. Curvature was calculated in Cartesian coordinates:

$$c=\frac{\left|x^{\prime }y^{\prime \prime }-y^{\prime }x^{\prime \prime }\right|}{{\left({x^{\prime }}^{2}+{y^{\prime }}^{2}\right)}^{\left(\frac{3}{2}\right)}}$$

where the derivatives were all calculated with respect to *t*, the distance along the tail.

### Extraction of global parameters

For all frames of a video, ZebraZoom outputs variables for each larva in each dish including: the position of its core, head axis, midline position of its tail and tail-bending angle. For each detected movement, a reference number for the larvae was extracted along with the corresponding well number, start and end time of the movement, and global parameters such as the number of oscillations, TBF, movement duration, heading direction range referring to the range of values of the heading axis for one movement with the heading angle reset to zero at the onset of movement, distance traveled, average speed (distance traveled divided by movement duration).

### Automatic multiclass categorization

We automatically attributed each movement detected in the video to either one of the three maneuvers: slow forward swim, routine turn or escape response. Our method relied on a dynamic set of parameters extracted from the bending angle of the tail estimated from the first tail bend over a limited time window (Figures 1C, 4A). We based our categorization on the four following parameters: (1) the amplitude of the tail-bending angle (0–178 ms, bins of 12 ms), (2) the instantaneous frequency (0–104 ms, bins of 7 ms), (3) the cumulative tail-bending angle calculated as the average angle value over time (0–178 ms, bins of 12 ms), and (4) the speed (0–240 ms, bins of 24 ms) (Figure 4A). The values of these four dynamic parameters were interpolated with a spline for a given time window during the movement and then used for categorization of every movement. PCA was first performed to reduce noise and dimensionality. Each movement was subsequently represented by the fourteen first principal components of the PCA out of 53 components (representing all together about 93% of the variance), to which the total duration of the movement was added. Multiclass categorization was implemented in two steps: a series of two subsequent SVM classifiers with linear kernel was applied for automatic categorization of movements: the first SVM classifier discriminated slow forward swims vs. turns and escapes, and if necessary a second SVM discriminated between a routine turn and an escape. We used two distinct datasets from WT 5–7 dpf larvae, one for learning the three maneuver types (five videos, *n* = 201 movements) and one for testing their recognition (three videos, *n* = 189 movements).

### Estimating the recurrence of maneuvers

Successions of maneuvers performed by larvae in a given dish were modeled as Markov chains. Out of the nine possible sequences of two maneuvers (S–S, S–T, S–E, T–S, T–T, T–E, E–S, E–T, E–E), we estimated the frequency of occurrence of each sequence. For a given movement classified as S, T, or E occurring at a given time in one dish, we calculated the transition probability for the subsequent movement to be classified as S, T, or E. We calculated a weighted transition index (*I*) for each sequence of two sequential maneuvers as the ratio of the transition probability from the first maneuver to the second, divided by the probability of occurrence of the second maneuver (Table 2 for values of all transition indexes). When *I* is equal to 1, the probability of repeating a maneuver is equal to the probability of random occurrence of the maneuver (probability of random occurrence was 0.35 for S; 0.48 for T and 0.16 for E; Table 2). Thus the index of recurrence *I* was defined as:

$$I(B1,B2)=\frac{p(x_{i}=B1|x_{i\;-1}=B2)}{p(x_{i}=B1)}$$

with *B*1 and *B*2 as two possible maneuvers (S, T, or E) and *x*_*i* − 1_ and *x*_*i*_ as two successive movements. WT larvae were used to estimate the transition index (36,068 movements from 280 larvae originating from four clutches and obtained from 40 wells). To investigate the recurrence of maneuvers as a function of time and distance, we calculated *I* as a function of the distance separating the two head centers of the larvae at the onset of their respective movement and the time as the time interval between the onsets of the first and second movement. *I* was calculated for many different time and distance windows. In Figure 5, we plotted these indexes for the sequences S–S, T–T, and E–E. We first calculated the index for the same larva (Figures 5Ai–iii) and across different larvae (Figures 5Bi–iii).

### Statistical analysis

The data used for Figure 2A were based on eight videos, 420 WT AB larvae from six different clutches between 5 and 7 dpf. All values were given as mean ± standard error of the mean (s.e.m.) calculated per movement. For the pharmacology experiments (Figure 2B), strychnine was bath applied at 75 μM and the data were based on two videos of 84 WT larvae coming from two clutches (42 for controls and 42 for strychnine) between 6 and 7 dpf. The data on *atoh7*^−/−^ mutants in Figure 2C were generated using four videos, 224 larvae total originating from four clutches (112 *atoh7*^−/−^ and 112 control siblings). All global parameters plotted in Figures 2B,C were calculated per larva then averaged across all larvae and means were given ± s.e.m. across all larvae. Since the distributions of global parameters were not normal, a standard non-parametric Wilcoxon rank sum test was used in MATLAB for calculating differences between conditions with vs. without drugs for Figure 2B and *atoh7*^−/−^ vs. siblings for Figure 2C. The data used for Figure 4D were based on 44,688 movements from eight videos, 448 larvae, six clutches and for Figure 5 from 36,068 movements from five videos, 280 WT AB larvae from four clutches. To test how the maximal values of the transition index were different from random, we calculated *I*_max_ after randomly permuting maneuvers while keeping track of the larva identity, time, and location the same for 50 iterations. For each comparison, S–S, T–T, E–E across different larvae or within the same larva, we compared the values of *I*_max_ after randomization to the measured value *I*_max_ using a two-sample *T*-test.

## Data and algorithm sharing

The software ZebraZoom is documented and available online from Source Forge in the code tab (http://sourceforge.net/p/zebrazoom/wiki/Home/). ZebraZoom requires MATLAB and works reliably on an Ubuntu 11.04 computer with OpenCV installed and MATLAB 7.10.

## Contributions

Claire Wyart and Olivier Mirat designed the project with help from Jenna R. Sternberg. Olivier Mirat wrote all algorithms of the ZebraZoom program. Jenna R. Sternberg and Claire Wyart assembled the experimental setup. Jenna R. Sternberg performed all experiments. Olivier Mirat and Claire Wyart analyzed the data. Kristen E. Severi, Olivier Mirat, and Claire Wyart prepared the figures. All authors tested and validated the program and wrote the paper.

### Conflict of interest statement

The authors declare that the research was conducted in the absence of any commercial or financial relationships that could be construed as a potential conflict of interest.

## Supplementary material

Video S1 **ZebraZoom tracking of seven larvae in a dish**. Acquisition was performed at 300 Hz, and one out of every ten images is displayed (every 33.3 ms).
